# New Synthetic 3-Benzoyl-5-Hydroxy-2*H*-Chromen-2-One (LM-031) Inhibits Polyglutamine Aggregation and Promotes Neurite Outgrowth through Enhancement of CREB, NRF2, and Reduction of AMPK*α* in SCA17 Cell Models

**DOI:** 10.1155/2020/3129497

**Published:** 2020-04-22

**Authors:** Chiung-Mei Chen, Wan-Ling Chen, Shu-Ting Yang, Te-Hsien Lin, Shu-Mei Yang, Wenwei Lin, Chih-Ying Chao, Yih-Ru Wu, Kuo-Hsuan Chang, Guey-Jen Lee-Chen

**Affiliations:** ^1^Department of Neurology, Chang-Gung Memorial Hospital, Chang Gung University College of Medicine, Taoyuan 33302, Taiwan; ^2^Department of Life Science, National Taiwan Normal University, Taipei 11677, Taiwan; ^3^Department of Chemistry, National Taiwan Normal University, Taipei 11677, Taiwan

## Abstract

Spinocerebellar ataxia type 17 (SCA17) is caused by a CAG/CAA expansion mutation encoding an expanded polyglutamine (polyQ) tract in TATA-box binding protein (TBP), a general transcription initiation factor. Suppression of cAMP-responsive element binding protein- (CREB-) dependent transcription, impaired nuclear factor erythroid 2-related factor 2 (NRF2) signaling, and interaction of AMP-activated protein kinase (AMPK) with increased oxidative stress have been implicated to be involved in pathogenic mechanisms of polyQ-mediated diseases. In this study, we demonstrated decreased pCREB and NRF2 and activated AMPK contributing to neurotoxicity in SCA17 SH-SY5Y cells. We also showed that licochalcone A and the related in-house derivative compound 3-benzoyl-5-hydroxy-2*H*-chromen-2-one (LM-031) exhibited antiaggregation, antioxidative, antiapoptosis, and neuroprotective effects in TBP/Q_79_-GFP-expressing cell models. LM-031 and licochalcone A exerted neuroprotective effects by upregulating pCREB and its downstream genes, BCL2 and GADD45B, and enhancing NRF2. Furthermore, LM-031, but not licochalcone A, reduced activated AMPK*α*. Knockdown of CREB and NRF2 and treatment of AICAR (5-aminoimidazole-4-carboxamide 1-*β*-D-ribofuranoside), an AMPK activator, attenuated the aggregation-inhibiting and neurite outgrowth promoting effects of LM-031 on TBP/Q_79_ SH-SY5Y cells. The study results suggest the LM-031 as potential therapeutics for SCA17 and probable other polyQ diseases.

## 1. Introduction

Hereditary spinocerebellar ataxia types (SCAs) 1, 2, 3, 6, 7, and 17 are among a group of inherited neurodegenerative diseases caused by the expansion of unstable trinucleotide (CAG) repeats encoding expanded polyglutamine (polyQ) tracts [1]. PolyQ-mediated diseases also include Huntington's disease (HD), spinobulbar muscular atrophy (SBMA), and dentatorubral-pallidoluysian atrophy (DRPLA). SCA17 is an autosomal dominant ataxia caused by an allele containing expanded repeats longer than 43 in the TATA-box binding protein (*TBP*) gene, a transcription initiation factor [2]. Protein containing an expanded polyQ tract tends to change conformation and form intranuclear and cytoplasmic aggregates, thereby causing several pathologic processes including transcriptional dysregulation, mitochondrial abnormalities, oxidative stress, axonal transport defect, chaperone-proteasome impairment, autophagolysosome dysfunction, and unfolded protein response (UPR) in the endoplasmic reticulum (ER) [1, 3]. Transcriptional dysregulation is one of the main pathogenic mechanisms of polyQ diseases [4]. Several lines of evidence have shown that nuclear localization of polyQ containing protein interferes with nuclear transcription factors and cofactors leading to cellular toxicity. CBP (cAMP-responsive element binding protein- (CREB-) binding protein), a cofactor for CREB-dependent transcriptional activation, has been shown to colocalize with the mutant polyQ containing protein in cell and mouse models and human HD brains [5]. Suppression of CREB-dependent transcription has been shown in a HD cell model, HD and SCA3 mice, and human HD brains [6–10]. Therefore, enhancement of CREB-mediated transcriptional activation is considered as a potential therapeutic strategy for polyQ diseases [11–16]. Since transcriptional dysregulation has been shown to play a role in pathogenic mechanisms of SCA17 [17], we proposed that CREB-mediated gene expression is downregulated in SCA17 and compounds augmenting CREB activation can rescue the cytotoxicity.

Increased oxidative stress induced by mutant polyQ protein, which results in cell death *in vitro* [18, 19] and *in vivo* [20, 21], has been shown. Antioxidants can ameliorate the aggregation and cytotoxicity in models of polyQ diseases [22–26]. The major pathway responsive to oxidative stress to protect cells is the nuclear factor erythroid 2-related factor 2 (NRF2) and the antioxidant response elements (AREs) signaling [27]. The target genes regulated by NRF2 are belonging to the endogenous phase II antioxidative enzymes. NRF2 activation can mitigate a number of neurodegenerative diseases including HD [28]. We and other researchers have shown that NRF2 expression is impaired in SCA3 and SCA17 models, and agents enhancing NRF2 rescue the phenotypes induced by mutant polyQ [2, 22, 29–32]. Taken together, we planned to examine more compounds that may activate NRF2 in our SCA17 cell models.

AMP-activated protein kinase (AMPK) is a serine/threonine kinase that plays a mandatory role in maintaining cellular metabolic homeostasis. AMPK is regulated by the cellular adenylate charge and is activated in response to energy deficiency in cells [33]. AMPK consists of three subunits (*α*, *β*, and *γ*) and is mainly activated through the phosphorylation of the threonine residue 172 (Thr172) of *α* subunit [34]. The activity of AMPK is regulated by several kinases including calmodulin-dependent protein kinase kinase (CaMKK), liver kinase B1 (LKB1), TGF-*β*-activated kinase 1 (TAK1), cAMP-dependent kinase (PKA), and Ca^2+^/calmodulin-dependent protein kinase II (CaMKII) [35]. The function of AMPK is multifold. The interactions between oxidative stress and AMPK are complex and may play a different role under different conditions. AMPK is activated to protect a rat dental pulp cell line under oxidative stress [36]. AMPK activation reverses decreased cell viability induced by ageing-related oxidative stress [37] and also protects cells from oxidative stress-induced senescence via autophagic flux restoration [38]. A previous study also has shown that mitochondrial reactive oxygen species (ROS) physiologically activate AMPK, which induce an antioxidant program that regulates mitochondrial homeostasis and cellular metabolic balance [39]. However, Ju and colleagues have shown that mutant huntingtin (mHTT) abnormally activates AMPK-*α*1 via a CaMKII-dependent pathway and exerts a detrimental effect on neuronal survival [40]. The authors further showed that increased ROS and the activated AMPK-*α*1 act in a vicious cycle contributing to the mHTT-induced neuronal death in the striatum of HD mice [41]. Since previously our SCA17 models displayed increased oxidative stress [29, 32, 42], we planned to investigate the role of AMPK in the pathogenesis of SCA17 and if aggregation-inhibitory compounds act on the AMPK pathway.

We have also previously shown that licochalcone A and five related in-house derivative LM compounds exhibited antiaggregation, antioxidant, and neuroprotective effects against A*β* toxicity by enhancing the NRF2-related antioxidant and CREB-dependent survival pathway [43]. Therefore, we tested the effects of licochalcone A and these LM compounds targeting these pathways in TBP/Q_79_-GFP-expressing cell models.

## 2. Materials and Methods

### 2.1. Compounds and Cell Culture

Licochalcone A was purchased from Sigma-Aldrich (St. Louis, MO, USA). In-house LM compounds LM-004, LM-006, LM-016, LM-026, and LM-031 were synthesized and characterized by NMR spectrum as described previously [43–45]. All compounds were soluble in a cell culture medium up to 100 *μ*M.

Human TBP/Q_79_-green fluorescent protein (GFP) 293 and SH-SY5Y cells [42] were maintained in Dulbecco's modified Eagle's medium (DMEM) (for 293 cells) or DMEM-F12 (for SH-SY5Y cells) supplemented with 10% fetal bovine serum (FBS) (Invitrogen, Carlsbad, CA, USA), with 5 *μ*g/mL blasticidin and 100 *μ*g/mL hygromycin (InvivoGen, San Diego, CA, USA) added to the growth medium.

### 2.2. Compound Cytotoxicity

Compound cytotoxicity was assessed by colorimetric assay measuring cell metabolic activity. Briefly, TBP/Q_79_-GFP 293 and SH-SY5Y cells were seeded on a 48-well plate (5 × 10^4^/well), grown for 20 h, and treated with licochalcone A or LM compounds (0.1–100 *μ*M). After one day, cell viability was measured based on reduction of 3,(4,5-dimethylthiazol-2-yl)-2,5-diphenyltetrazolium bromide (MTT), and the absorbance of the insoluble purple formazan product at OD 570 nm was read by a FLx800 fluorescence microplate reader (Bio-Tek, Winooski, VT, USA). Half-maximal inhibitory concentration (IC_50_) was defined as the concentration of compounds required for the reduction of 570 nm signals by 50%.

### 2.3. TBP/Q_79_ 293 Aggregation Assay

GFP fluorescence was evaluated to reflect TBP aggregation in TBP/Q_79_-GFP-expressing 293 cells. Briefly, cells were plated on 96-well (2 × 10^4^/well) dishes, grown for 24 h and treated with different concentrations of licochalcone A or LM compounds (0.1 nM−100 *μ*M) or a positive control suberoylanilide hydroxamic acid (SAHA, 100 nM) [46] (Cayman Chemical, Ann Arbor, MI, USA). After 8 h, doxycycline (10 *μ*g/mL) was added to the cells for 6 days to induce TBP/Q_79_-GFP expression. In addition, oxaliplatin (5 *μ*M) (Sigma-Aldrich, St Louis, MO, USA) was included for TBP/Q_79_-GFP aggregate accumulation via blocking cell-cycle progression [47]. On the eighth day, the cells were stained with Hoechst 33342 (0.1 *μ*g/mL; Sigma-Aldrich) for 30 min, and cell images were automatically recorded (excitation/emission wavelengths of 482/536 nm for GFP and 377/447 nm for Hoechst 33342) by using an ImageXpress Micro Confocal High-Content Imaging System (Molecular Devices, Sunnyvale, CA, USA). Aggregation was determined by a Transfluor technology [48] based on GFP fluorescence intensity. To quantify aggregation, the relative aggregation level in untreated cells was set as 100%. Aggregation was measured in wells containing ≥80% viable cells.

### 2.4. Reactive Oxygen Species Analysis

TBP/Q_79_-GFP 293 cells were seeded on a 6-well plate (5 × 10^4^/well) and treated with licochalcone A or LM compound (100 nM); then, TBP/Q_79_-GFP expression was induced as described. On day 8, CellROX™ Deep Red (Molecular Probes, Waltham, MA, USA) was added to a final concentration of 5 *μ*M and incubated at 37°C for 30 min. Reactive oxygen species (ROS) in cells were measured using a flow cytometer (Becton Dickinson, Franklin Lakes, NJ, USA) with excitation/emission wavelengths at 488/507 nm (green, TBP/Q_79_-GFP expression) and 640/665 nm (red, ROS). For each sample, 5 × 10^4^ cells were analyzed.

### 2.5. TBP/Q_79_-GFP SH-SY5Y Aggregation and Neurite Outgrowth Assays

On day 1, 2 × 10^4^ of SH-SY5Y TBP/Q_79_-GFP cells were seeded on a 24-well plate with retinoic acid (10 *μ*M; Sigma-Aldrich) added to induce neuronal differentiation [49]. On day 2, the cells were treated with LM-031 or licochalcone A (100 nM) for 8 h and TBP/Q_79_-GFP expression was induced as described. On day 8, cells were stained with Hoechst 33342 (0.1 *μ*g/mL) and the aggregation percentage and neurite outgrowth were assessed by high-content analysis as described. The morphologic differentiation of TBP/Q_79_-GFP-expressing cells was analyzed by using the Neurite Outgrowth Application Module (Molecular Devices).

### 2.6. Caspase 3 Activity Assay

As described, TBP/Q_79_-GFP SH-SY5Y cells were seeded on a 12-well plate (1 × 10^5^/well) with retinoic acid added on day 1; treatment with LM-031 or licochalcone A (100 nM) and induction of TBP/Q_79_-GFP followed on day 2. On day 8, cells were lysed in 1 × lysis buffer by repeated cycles of freezing and thawing. Caspase 3 activity was measured with the caspase 3 assay kit according to the manufacturer's instructions (Sigma-Aldrich).

### 2.7. Western Blot Analysis

Total proteins from TBP/Q_79_-GFP SH-SY5Y cells were obtained using a lysis buffer containing 50 mM Tris-HCl (pH 8.0), 150 mM NaCl, 1 mM EDTA (pH 8.0), 1 mM EGTA (pH 8.0), 0.1% SDS, 0.5% sodium deoxycholate, 1% Triton X-100, and a protease inhibitor cocktail (Sigma-Aldrich). After quantitation using a protein assay kit (Bio-Rad, Hercules, CA, USA), proteins (20 *μ*g) were separated on 10−12% SDS-polyacrylamide gel electrophoresis and blotted onto polyvinylidene difluoride (PVDF) membranes (Sigma-Aldrich) by reverse electrophoresis. After blocking, the membrane was probed with CREB (1 : 1000; Santa Cruz Biotechnology, Santa Cruz, CA, USA), pCREB (S133) (1 : 1000; Santa Cruz Biotechnology), BCL2 (1 : 500; BioVision, Milpitas, CA, USA), GADD45B (1 : 1000; Abcam, Cambridge, MA, USA), BAX (1 : 500; BioVision), NRF2 (1 : 500; Santa Cruz Biotechnology), AMPK*α* (1 : 1000; Cell Signaling, Danvers, MA, USA), pAMPK*α* (T172) (1 : 1000; Cell Signaling), GAPDH (1 : 1000) (MDBio Inc., Taipei, Taiwan), or *β*-actin (1 : 5000; Millipore, Billerica, MA, USA) primary antibody at 4°C overnight. The immune complexes were detected using a horseradish peroxidase-conjugated goat anti-mouse or goat anti-rabbit IgG antibody (1 : 10000; GeneTex, Irvive, CA, USA) and a chemiluminescent substrate (Millipore).

### 2.8. RNA Interference

To knockdown the expression of NRF2 and CREB in TBP/Q_79_-GFP SH-SY5Y cells, lentiviruses with short hairpin RNA (shRNA) targeting NRF2 (TRCN0000007558), CREB (TRCN0000226466), and a negative control scrambled (TRC2.Void) were obtained from the National RNAi Core Facility, Institute of Molecular Biology/Genomic Research Center, Academia Sinica, Taipei, Taiwan. On day 1, cells were plated on 6-well plates (8 × 10^5^/well for protein analysis) or 24-well plates (2 × 10^4^/well for neurite outgrowth analysis) in the presence of retinoic acid as described. On day 2, the cells were infected with lentivirus (multiplicity of infection: 3) in the presence of polybrene (8 *μ*g/mL; Sigma-Aldrich) to increase infectivity. On day 3, the cells were pretreated with LM-031 or licochalcone A (100 nM) for 8 h; induction of TBP/Q_79_-GFP expression followed. On day 9, the cells were collected for NRF2 and CREB protein analysis or stained with Hoechst 33342 and analyzed for aggregation and neurite outgrowth as described.

### 2.9. AICAR Treatment

AICAR (5-aminoimidazole-4-carboxamide 1-*β*-D-ribofuranoside) (Sigma-Aldrich), an analog of adenosine monophosphate (AMP), was used to stimulate AMP-dependent protein kinase (AMPK) activity. TBP/Q_79_-GFP SH-SY5Y cells were plated on 6-well or 24-well plates in the presence of retinoic acid as described. On day 2, the cells were pretreated with LM-031 or licochalcone A (100 nM) for 4 h; AICAR (0.1 mM) treatment and TBP/Q_79_-GFP induction followed. On day 8, the cells were collected for AMPK*α* and pAMPK*α* protein analysis or stained with Hoechst 33342 and analyzed for aggregation and neurite outgrowth as described.

### 2.10. Trx- and His-Tagged TBP/Q_20-61_ and Thioflavin T Binding/Filter Trap Assays

TBP cDNA containing 20 or 61 combined repeats was generated by ligating *Fnu*4HI partially digested fragments as described [50]. PCR was performed using the cloned TBP/Q_20−61_ as templates and synthetic primers 5′-GAACACCATGGATCAGAACAACAGCCTGCCAC (*Nco*I site underlined) and 5′-GGCTCGAGTGGCGTGGCAGGAGTGATGGGGGTC (*Xho*I site underlined). The amplified polyQ-containing cDNA fragments were cloned into pGEM-T Easy (Promega, Fitchburg, WI, USA) and sequenced. The TBP/Q_20−61_ cDNAs were excised with *Nco*I and *Xho*I and subcloned into the corresponding sites of pET-32b(+) (Novagen, Madison, WI, USA). The resulting plasmids were transformed into BL21(DE3)pLysS (Novagen), and Trx- (thioredoxin-) and His-tagged TBP/Q_20−61_ protein expressions were induced with 0.1 mM isopropyl-*β*-D-thiogalactopyranoside (IPTG) for 3 h at 37°C. Bacterial cells were then harvested, and the Trx-His-TBP/Q_20−61_ proteins were purified using His-Bind resins (Novagen) and verified by probing with the TBP antibody (1 : 1000; Santa Cruz Biotechnology).

For thioflavin T binding assay, TBP protein (5 *μ*g in final 200 *μ*L) was incubated with tested compounds (congo red, 1−10 *μ*M; LM-031 and licochalcone A, 1−100 *μ*M) in 150 mM NaCl and 20 mM Tris-HCl, pH 8.0, at 37°C for 24 h to form aggregates. Then, thioflavin T (20 *μ*M final concentration; Sigma-Aldrich) was added and incubated for 30 min at room temperature. Thioflavin T fluorescence intensity of samples was recorded by using a microplate reader (Bio-Tek FLx800), with excitation 420 nm and emission 485 nm filter combination.

For filter trap assay to quantify polyQ aggregates, the purified TBP protein was incubated with tested compound (100 *μ*M) at 37°C for 24 h as described previously. In brief, protein (1 *μ*g) was diluted in 2% SDS in PBS and filtered through a cellulose acetate membrane (0.2 *μ*m pore size; Merck, Kenilworth, NJ, USA) preequilibrated in 2% SDS in PBS on a dot-blot filtration unit (Bio-Rad Laboratories, Hercules, CA, USA). After three washes with 2% SDS buffer, the membrane was blocked in PBS containing 5% nonfat dried milk and stained with anti-TBP antibody (1 : 1000; Santa Cruz Biotechnology). The immune complexes containing aggregates on the filter were detected as described.

### 2.11. Statistical Analysis

For each data set, the experiments are performed three times and data were expressed as the means ± standard deviation (SD). Differences between groups were evaluated using Student's *t* test (comparing two groups) or one-way analysis of variance with a *post hoc* LSD test where appropriate (comparing several groups). *p* values lower than 0.05 were considered statistically significant.

## 3. Results

### 3.1. Test Compounds and IC_50_ Cytotoxicity

**Figure 1:**
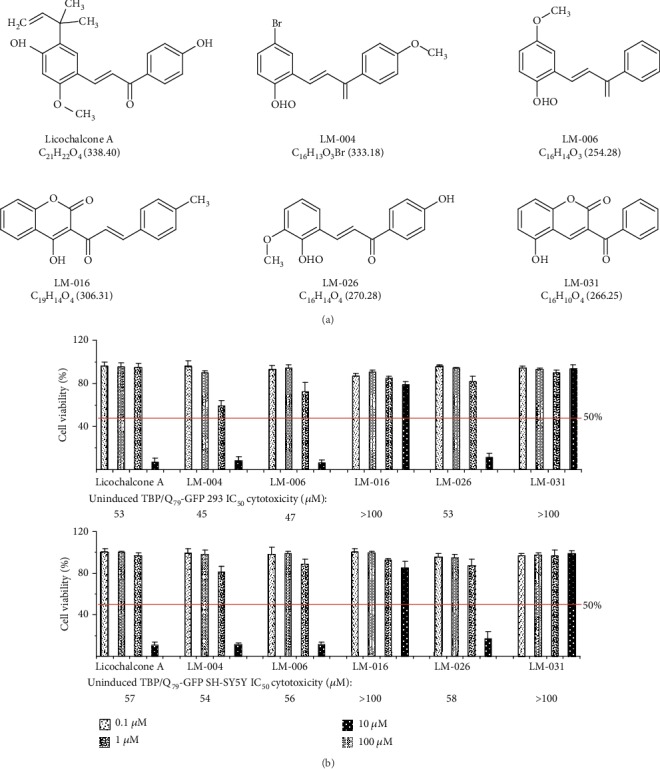
Test compounds and cytotoxicity. (a) Structure, formula, and molecular weight of licochalcone A, LM-004, LM-006, LM-016, LM-026, and LM-031. (b) Cytotoxicity of test compounds (0.1−100 *μ*M) in uninduced TBP/Q_79_-GFP 293 and SH-SY5Y cells using the MTT assay (*n* = 3). To normalize, the relative untreated cell viability was set as 100%. Values shown are the IC_50_ values.

Licochalcone A and five related in-house LM compounds were tested (Figure 1(a)). The MTT assay was performed using uninduced TBP/Q_79_-GFP 293 and SH-SY5Y cells following treatment with the test compounds (0.1−100 *μ*M) for 24 h. The IC_50_ values of licochalcone A, LM-004, LM-006, LM-016, LM-026, and LM-031 in uninduced 293/SH-SY5Y cells were 53/57, 45/54, 47/56, >100/>100, 53/58, and >100/>100 *μ*M, respectively (Figure 1(b)). The results demonstrated the low cytotoxicity of test compounds in TBP/Q_79_-GFP 293 and SH-SY5Y cells, especially LM-031.

### 3.2. Reduction of TBP/Q_79_ Aggregation and Oxidative Stress of Licochalcone A and LM Compounds in SCA17 293 Cell Model

**Figure 2:**
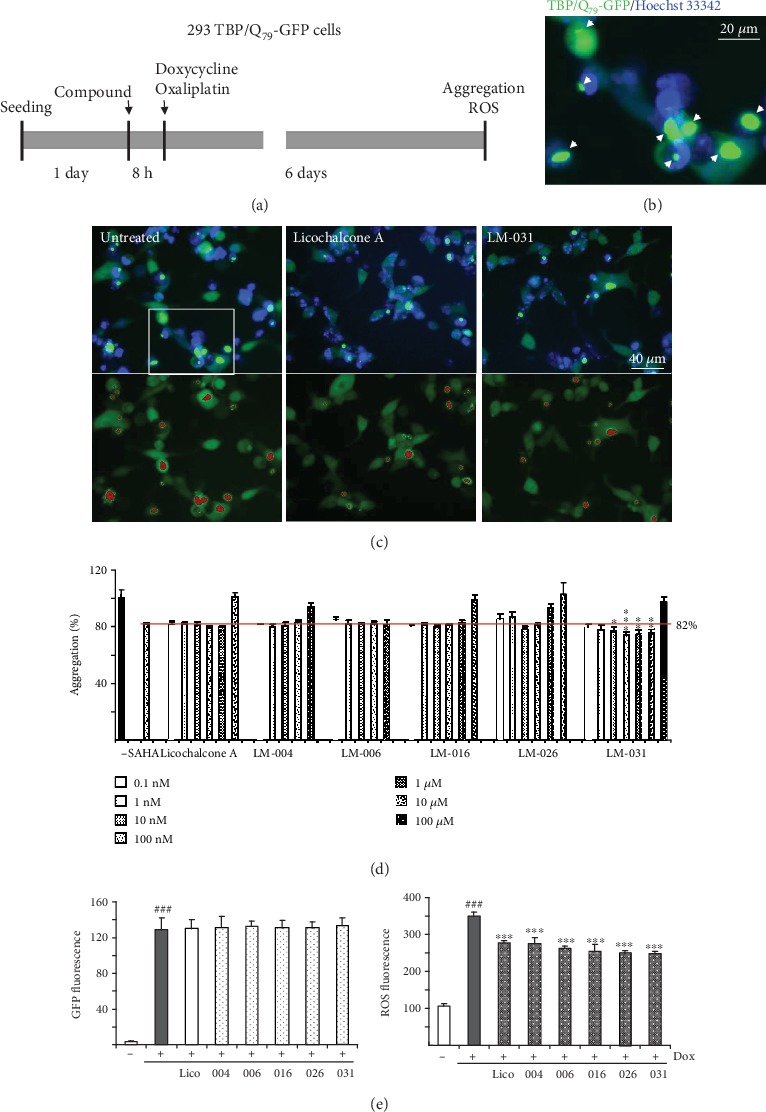
Aggregation and ROS analyses on TBP/Q_79_-GFP-expressing 293 cells. (a) Experimental flow chart. TBP/Q_79_-GFP 293 cells were plated on dishes, grown for 24 h, and treated with SAHA (100 nM) or test compounds (0.1 nM−100 *μ*M) for 8 h. Then, doxycycline (10 *μ*g/mL) and oxaliplatin (5 *μ*M) were added to the medium for 6 days, followed by aggregation (by HCA) and ROS (by flow cytometry) measurements. (b) Fluorescent microscopy image of cells with induced TBP/Q_79_-GFP expression (green) for six days. Nuclei were counterstained with Hoechst 33342 (blue). White arrows indicate aggregates. (c) Representative microscopy images of TBP/Q_79_-GFP cells untreated, or treated with licochalcone A or LM-031 (100 nM) for 6 days, with nuclei counterstained (blue, top row) or aggregates marked (red, bottom row). The magnified boxed area of the untreated TBP/Q_79_-GFP-expressing 293 cells is displayed as (b). (d) Aggregation analysis (*n* = 3) of TBP/Q_79_-GFP-expressing cells untreated or treated with SAHA (100 nM) or test compounds (0.1 nM−100 *μ*M). To normalize, the relative aggregation level in untreated cells was set as 100%. The red line represents 82% aggregation for 100 nM SAHA treatment. *p* values: comparisons between test-compounds treated and SAHA treated (^∗^*p* < 0.05, ^∗∗^*p* < 0.01, ^∗∗∗^*p* < 0.001). Aggregation was analyzed in wells containing at least 80% viable cells. (e) The induced GFP and ROS levels were measured by flow cytometry (*n* = 3). *p* values: comparisons between induced and uninduced cells (^###^*p* < 0.001), or between compound (100 nM) treated and untreated cells (^∗∗∗^*p* < 0.001).

To examine the polyQ aggregation-inhibitory and ROS-reducing effects of test compounds, TBP/Q_79_-GFP 293 cells were treated with licochalcone A, LM compounds (0.1 nM–100 *μ*M), or histone deacetylase inhibitor SAHA (100 nM) for 8 h and induced TBP/Q_79_-GFP expression (by doxycycline) under cell division inhibition (by oxaliplatin) for 6 days (Figure 2(a)). While no TBP/Q_79_-GFP expression was seen in the absence of doxycycline (data not shown), TBP/Q_79_-GFP aggregates were readily seen in the induced cells (Figure 2(b)). Representative microscopy images of TBP/Q_79_-GFP aggregation in untreated cells or after licochalcone A or LM-031 treatment (100 nM) were shown in Figure 2(c). As a positive control, SAHA at 100 nM significantly reduced the TBP/Q_79_-GFP aggregation to 82% (*p* < 0.001) compared with untreated cells (100%) (Figure 2(d)). Treatment of licochalcone A (0.1 nM–1 *μ*M), LM-004 (0.1 nM–100 nM), LM-006 (0.1 nM–1 *μ*M), LM-016 (0.1 nM–1 *μ*M), LM-026 (0.1 nM–100 nM), and LM-031 (0.1 nM–10 *μ*M) also significantly reduced the TBP/Q_79_-GFP aggregation (87–74%, *p* < 0.001). In addition, aggregation-inhibitory effect of LM-031 at 10 nM–10 *μ*M was significantly better than that of SAHA at 100 nM (74–76%, *p* = 0.027−<0.001).

Abnormal TBP-containing polyQ expansion has been shown to increase cellular ROS level [42]. To evaluate whether licochalcone A or LM compounds reduced oxidative stress in TBP/Q_79_-GFP 293 cells, the cellular ROS production was measured. As shown in Figure 2(e), significantly increased ROS production (179% of control, *p* = 0.001) was observed in cells with induced TBP/Q_79_-GFP expression (+Dox) for 6 days (33.8-fold expression, *p* < 0.001). With the similar induced green fluorescence (34.1–34.9-fold, *p* > 0.05), the test licochalcone A and LM compounds (100 nM) significantly ameliorated oxidative stress induced by TBP/Q_79_-GFP expression (ROS fluorescence: from 349 to 277–247, *p* < 0.001).

### 3.3. Neuroprotective Effects of LM-031 and Licochalcone A in SCA17 SH-SY5Y Cell Model

**Figure 3:**
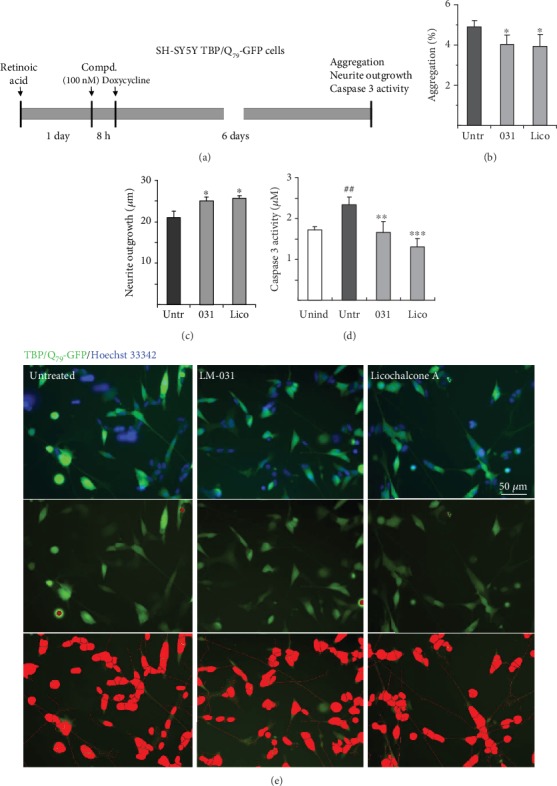
Neuroprotective effects of LM-031 and licochalcone A in TBP/Q_79_-GFP-expressing SH-SY5Y cells. (a) Experimental flow chart. TBP/Q_79_-GFP SH-SY5Y cells were plated on dishes with retinoic acid (10 *μ*M) added on day 1 to initiate neuronal differentiation. On the next day, LM-031 or licochalcone A (100 nM) was added to the cells for 8 h followed by inducing TBP/Q_79_-GFP expression with doxycycline (5 *μ*g/mL) for 6 days. Aggregation, neurite outgrowth (by HCA), and caspase 3 activity were assessed. (b) Relative aggregation, (c) neuronal outgrowth, and (d) caspase 3 activity of TBP/Q_79_-GFP-expressing SH-SY5Y cells with LM-031 or licochalcone A treatment (*n* = 3). *p* values: comparisons between treated and untreated cells (^∗^*p* < 0.05, ^∗∗^*p* < 0.01, ^∗∗∗^*p* < 0.001) or between untreated and uninduced cells (^##^*p* < 0.01). (e) Representative microscopy images of differentiated TBP/Q_79_-GFP-expressing SH-SY5Y cells untreated or treated with LM-031 or licochalcone A, with nuclei counterstained in blue (top row), aggregates marked in red (middle row), or neurites and cell bodies outlined in red for outgrowth quantification (bottom row).

To further examine the aggregation-reducing and neurite outgrowth-promoting potentials of LM-031 and licochalcone A in neuronal cells, TBP/Q_79_-GFP SH-SY5Y cells were differentiated using retinoic acid for 8 days (Figure 3(a)). Treatment with LM-031 or licochalcone A (100 nM) led to 18–20% reduction of aggregation in TBP/Q_79_-GFP-expressing neuronal cells (from 4.9% to 4.0–3.9%, *p* = 0.018 − 0.015; Figure 3(b)). In addition, significantly increased neurite outgrowth (19−22%) was observed with LM-031 or licochalcone A treatment (from 21.1 *μ*m to 25.1–25.6 *μ*m, *p* = 0.022 − 0.019; Figure 3(c)). Moreover, pretreatment with LM-031 or licochalcone A significantly reduced the caspase 3 activity compared to no treatment (from 2.3 *μ*M to 1.7−1.3 *μ*M%, *p* = 0.002−<0.001; Figure 3(d)). Representative images of TBP/Q_79_-GFP cells untreated or treated with LM-031 or licochalcone A (100 nM) are shown in Figure 3(e). These results demonstrate the neuroprotective effects of LM-031 and licochalcone A in differentiated neurons expressing TBP/Q_79_-GFP.

### 3.4. Molecular Targets of LM-031 and Licochalcone A in SCA17 SH-SY5Y Cell Model

**Figure 4:**
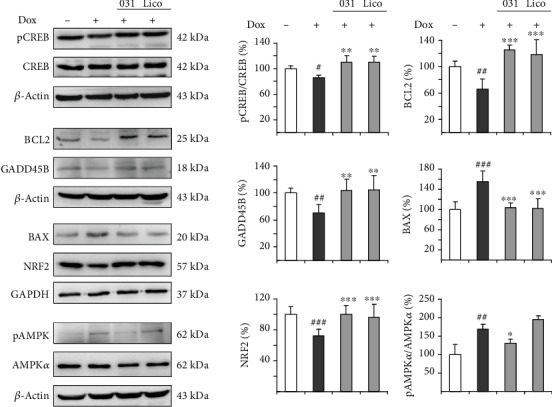
Expression of CREB, NRF2, and AMPK*α* following LM-031 or licochalcone A administration on TBP/Q_79_-GFP-expressing SH-SY5Y cells. On day 2, differentiated SH-SY5Y cells were pretreated with 100 nM LM-031 or licochalcone A for 8 h and TBP/Q_79_-GFP expression was induced for 6 days. Relative CREB, pCREB, BCL2, GADD45B, BAX, NRF2, and AMPK*α* and pAMPK*α* protein levels were analysed by immunoblot using *β*-actin or GAPDH as a loading control (*n* = 3). To normalize, expression level in uninduced (without Dox) cells was set at 100%. *p* values: comparisons between induced and uninduced cells (^#^*p* < 0.05, ^##^*p* < 0.01, ^###^*p* < 0.001) or between treated and untreated cells (^∗^*p* < 0.05, ^∗∗^*p* < 0.01, ^∗∗∗^*p* < 0.001).

CREB is a transcription factor binding to cAMP-responsive elements to promote transcription of BCL2 (BCL2 apoptosis regulator) [51] and GADD45B (growth arrest and DNA damage-inducible beta) [52, 53] genes for neuronal survival. Phosphorylation at Ser-133 induces CREB-mediated transcription of BCL2 and GADD45B. NRF2 is a master regulator of the antioxidant response through regulating the expression of phase II antioxidant and detoxification genes [27]. Both CREB and NRF2 have been shown to suppress polyQ-mediated toxicity in *Drosophila* [12, 31]. As the neuroprotective effects of LM-031 and licochalcone A in A*β*-GFP-expressing SH-SY5Y cells had been linked to CREB and NRF2 pathways [43], we examined the expressions of CREB, pCREB (S133), BCL2, GADD45B, and NRF2 by immunoblotting using specific antibodies. Induced expression of TBP/Q_79_-GFP in differentiated SH-SY5Y cells significantly attenuated phosphor/total ratio of CREB (85% of control, *p* = 0.044) and the downstream BCL2 and GADD45B (66–70% of control, *p* = 0.003–0.002). This attenuation was rescued by the treatment with LM-031 and licochalcone A: increased to 110% for pCREB/CREB (*p* = 0.005), 125–118% for BCL2 (*p* < 0.001), and 104–105% for GADD45B (*p* = 0.001) (Figure 4). In response to the antiapoptotic BCL2 change, the addition of LM-031 and licochalcone A significantly reduced the expression of proapoptotic BAX (BCL2 associated X, apoptosis regulator) (from 156% to 103%, *p* < 0.001). In addition, induction of TBP/Q_79_-GFP significantly reduced NRF2 expression (72% of control, *p* < 0.001). This reduction was also rescued by the treatment with LM-031 and licochalcone A (increased to 96–100%, *p* < 0.001) (Figure 4).

AMPK is a major energy sensor that maintains cellular energy homeostasis in the brain [54]. In Huntington's disease, mutant huntingtin protein induces oxidative stress to lead to aberrant activation of AMPK*α*1 and neuronal atrophy [40, 41]. As increased ROS was associated with TBP/Q_79_-GFP expression (Figure 2(e)), we examined the effects of LM-031 and licochalcone A on the alternation of AMPK. As also shown in Figure 4, phosphor/total ratio of AMPK*α* was significantly increased after induction of TBP/Q_79_-GFP expression (170% of control, *p* = 0.003), whereas treatment with LM-031, but not licochalcone A, reduced the phosphor/total ratio of AMPK*α* (from 170% to 130%, *p* = 0.034).

### 3.5. NRF2, CREB, and AMPK*α* as Therapeutic Targets in SCA17 SH-SY5Y Cell Model

**Figure 5:**
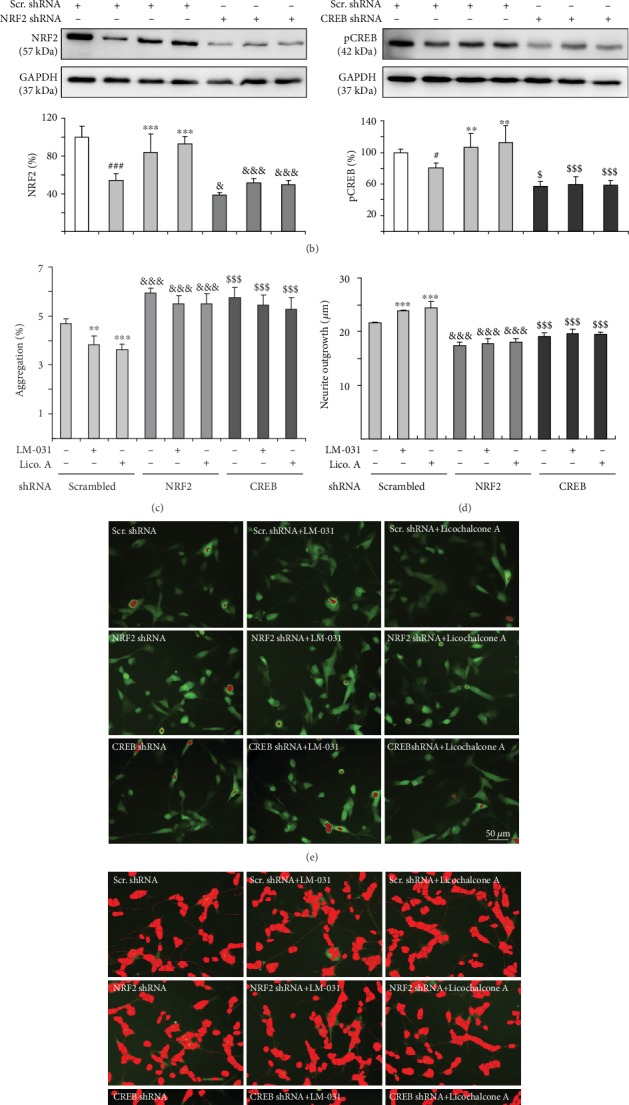
NRF2 and CREB as therapeutic targets in LM-031 and licochalcone A-treated TBP/Q_79_-GFP-expressing SH-SY5Y cells. (a) Experimental flow chart. On day 1, TBP/Q_79_-GFP SH-SY5Y cells were plated with retinoic acid (10 *μ*M). On day 2, the cells were infected with lentivirus-expressing NRF2-specific, CREB-specific, or scrambled shRNA (MOI: 3). At 24 h postinfection, LM-031 or licochalcone A (100 nM) was added to the cells for 8 h, followed by induction of TBP/Q_79_-GFP expression (Dox, 5 *μ*g/mL) for 6 days. On day 9, the cells were collected for NRF2 and pCREB protein analysis (by Western blot, GAPDH as a loading control) or stained with Hoechst 33342 for aggregation and neurite outgrowth analyses (by HCA). (b) Western blot analysis of NRF2 and pCREB protein levels in LM-031 or licochalcone A-treated cells infected with NRF2, CREB, or a negative control scramble (Scr.) shRNA. To normalize, the relative NRF2 or pCREB level of scramble shRNA-infected cells without inducing TBP/Q_79_-GFP expression was set as 100%. *p* values: comparisons between induced versus uninduced cells (^#^*p* < 0.05, ^###^*p* < 0.001), compound-treated versus untreated cells (^∗∗^*p* < 0.01, ^∗∗∗^*p* < 0.001), or scramble versus NRF2 or CREB shRNA-infected cells (^&^ or ^$^: *p* < 0.05, ^&&&^ or ^$$$^: *p* < 0.001) (*n* = 3). (c) Aggregation and (d) neurite outgrowth assays of LM-031 or licochalcone A-treated TBP/Q_79_-GFP SH-SY5Y cells infected with NRF2, CREB, or a scramble shRNA. *p* values: comparisons between compound-treated versus untreated cells (^∗∗^*p* < 0.01, ^∗∗∗^*p* < 0.001) or scramble shRNA versus NRF2 or CREB shRNA-infected cells (^&&&^ or ^$$$^: *p* < 0.001) (*n* = 3). (e−f) Representative microscopy images of TBP/Q_79_-GFP-expressing SH-SY5Y cells infected with scrambled (Scr.), NRF2, or CREB shRNA, or LM-031 and licochalcone A (100 nM)-treated cells infected with scrambled, NRF2, or CREB shRNA. Aggregates were marked in red (e), and neurites and cell bodies were outlined (f) for outgrowth quantification.

To validate the potential of NRF2 and CREB as therapeutic targets of LM-031 and licochalcone A, we knocked down NRF2 and CREB expression through lentivirus-mediated shRNA targeting of NRF2 and CREB in TBP/Q_79_-GFP-expressing SH-SY5Y cells (Figure 5(a)). In scrambled shRNA-infected cells, induced expression of TBP/Q_79_-GFP reduced the expression of NRF2 (54% of control, *p* < 0.001) and pCREB (81% of control, *p* = 0.037), and treatment with LM-031 or licochalcone A significantly increased NRF2 (from 54% to 84–93%, *p* < 0.001) and pCREB (from 81% to 107–112%, *p* = 0.007–0.002) levels (Figure 5(b)). Compared with scrambled control shRNA, NRF2 and CREB shRNA attenuated NRF2 and pCREB levels, respectively, in TBP/Q_79_-GFP-expressing cells without LM-031/licochalcone A treatment (NRF2: from 54% to 39%, *p* = 0.030; pCREB: from 81% to 57%, *p* = 0.014) or with LM-031/licochalcone A treatment (NRF2: from 84–93% to 51–50%, *p* < 0.001; pCREB: from 107–112% to 60–58%, *p* < 0.001) (Figure 5(b)). In line with NRF2 or pCREB expression, the reduced level of aggregation after treatment with LM-031 or licochalcone A (scrambled: 3.8–3.6%, *p* = 0.001–<0.001 compared with no treatment: 4.7%) was significantly increased by NRF2 or CREB shRNA (NRF2 shRNA: 5.5%, *p* < 0.001; CREB shRNA: 5.4–5.3%, *p* < 0.001) (Figure 5(c)). Furthermore, improvement in neurite outgrowth by LM-031 or licochalcone A treatment (scrambled: 23.8–24.6 *μ*m, *p* < 0.001 compared with no treatment: 21.6 *μ*m) was significantly attenuated by NRF2 or CREB shRNA knockdown (NRF2 shRNA: 17.8–18.1 *μ*m, *p* < 0.001; CREB shRNA: 19.6–19.5 *μ*m, *p* < 0.001) (Figure 5(d)). Representative images of scrambled/NRF2/CREB shRNA-infected TBP/Q_79_-GFP cells untreated or treated with LM-031 or licochalcone A are shown in Figures 5(e)–5(f). These results suggested that LM-031 and licochalcone A exerted neuroprotective effects by upregulating NRF2 and pCREB expression.

**Figure 6:**
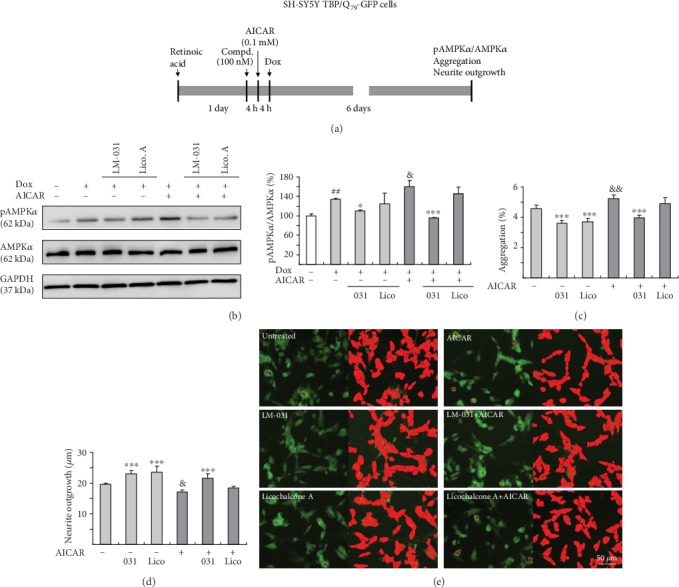
AMPK*α* as a therapeutic target in LM-031-treated TBP/Q_79_-GFP-expressing SH-SY5Y cells. (a) Experimental flow chart. TBP/Q_79_-GFP SH-SY5Y cells were plated with retinoic acid (10 *μ*M) added on day 1. The next day, LM-031 or licochalcone A (100 nM) was added to the cells. At 4 h postcompound treatment, AICAR (0.1 mM) was added to the cells for 4 h followed by inducing TBP/Q_79_-GFP expression (Dox, 5 *μ*g/mL) for 6 days. On day 9, the cells were collected for AMPK*α* and pAMPK*α* protein analysis (by Western blot, GAPDH as a loading control) or stained with Hoechst 33342 for aggregation and neurite outgrowth analyses (by HCA). (b) Western blot analysis of AMPK*α* and pAMPK*α* protein levels in LM-031 or licochalcone A-treated cells treated with AICAR. To normalize, the relative AMPK*α* or pAMPK*α* level of AICAR-untreated cells without inducing TBP/Q_79_-GFP expression was set as 100%. *p* values: comparisons between induced versus uninduced cells (^##^*p* < 0.01), compound-treated versus untreated cells (^∗^*p* < 0.05, ^∗∗∗^*p* < 0.001), or AICAR-treated versus untreated cells (^&^*p* < 0.05) (*n* = 3). (c) Aggregation and (d) neurite outgrowth assays of LM-031 or licochalcone A-treated TBP/Q_79_-GFP SH-SY5Y cells treated with AICAR. *p* values: comparisons between compound-treated versus untreated cells (^∗∗∗^*p* < 0.001) or AICAR-treated versus untreated cells (^&^*p* < 0.05, ^&&^*p* < 0.01) (*n* = 3). (e) Representative microscopy images of TBP/Q_79_-GFP-expressing SH-SY5Y cells treated with LM-031 or licochalcone A, and AICAR-treated cells treated with LM-031 or licochalcone A. Aggregates were marked in red (left) and neurites and cell bodies were outlined (right) for outgrowth quantification.

In addition, we explored the effects of AMPK*α* activation in aggregation and neurite outgrowth via AICAR (AMPK activator) administration (Figure 6(a)). Induced expression of TBP/Q_79_-GFP increased phosphor/total ratio of AMPK*α* (134% of control, *p* = 0.003) and treatment with LM-031, but not licochalcone A, significantly reduced pAMPK*α* level (from 134% to 110%, *p* = 0.021) (Figure 6(b)). AICAR treatment further raised pAMPK*α* level in TBP/Q_79_-GFP-expressing cells (from 134% to 160%, *p* = 0.013) and LM-031 treatment reduced pAMPK*α* level (from 160% to 96%, *p* < 0.001). In TBP/Q_79_-GFP-expressing SH-SY5Y cells, both LM-031 and licochalcone A reduced polyQ aggregation (3.6–3.7%, *p* < 0.001 compared with no treatment: 4.6%) (Figure 6(c)) and increased neurite outgrowth (23.0–23.6 *μ*m, *p* < 0.001 compared with no treatment: 19.6 *μ*m) (Figure 6(d)). AICAR treatment increased aggregation (5.2%, *p* = 0.004) and impaired neurite outgrowth (17.2 *μ*m, *p* = 0.010). LM-031 also reduced polyQ aggregation (from 5.2% to 4.0%, *p* < 0.001) and improved neurite outgrowth (from 17.2 *μ*m to 21.6 *μ*m, *p* < 0.001) in AICAR-treated TBP/Q_79_-GFP-expressing cells. However, levels of aggregation (from 5.2% to 4.9%, *p* > 0.05) and neurite outgrowth (from 17.2 *μ*m to 18.4 *μ*m, *p* > 0.05) were not significantly affected with licochalcone A treatment. Representative images of untreated/AICAR-treated TBP/Q_79_-GFP cells untreated or treated with LM-031 or licochalcone A are shown in Figure 6(e). The results showed that the beneficial effect of LM-031 was not significantly attenuated by AMPK activator AICAR.

### 3.6. Effects of LM-031 and Licochalcone A on polyQ Aggregation by Thioflavin T Fluorescence and Filter Trap Assays

**Figure 7:**
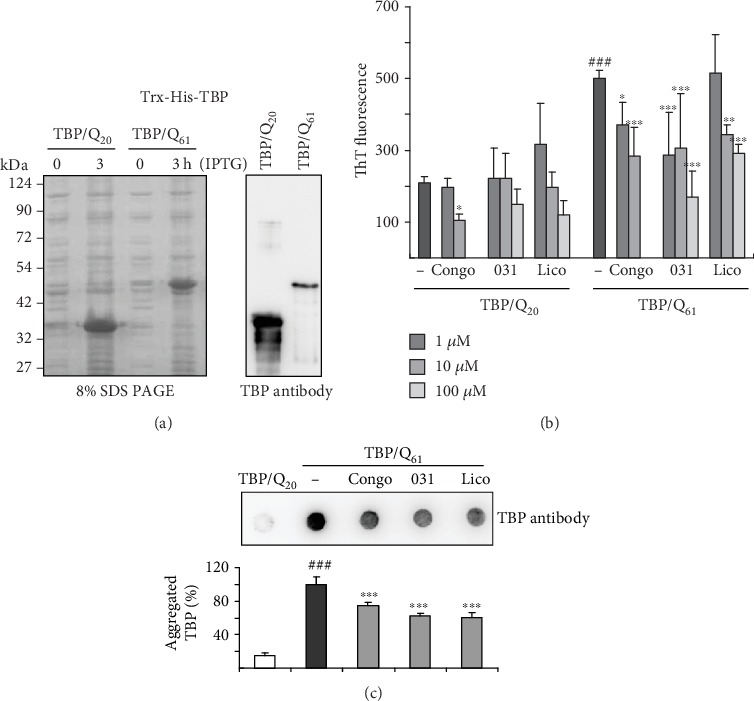
Trx-His-TBP/Q_20−61_ proteins and TBP aggregation monitored by thioflavin T fluorescence and filter trap assays. (a) SDS-PAGE gel of Trx-His-TBP/Q_20−61_ proteins from IPTG-induced bacterial cells (left) and Western blot analysis of purified TBP proteins using anti-TBP antibody (right). (b) Thioflavin T binding assay for Trx-His-TBP aggregation. TBP protein (5 *μ*g in 200 *μ*L PBS) was incubated with congo red (1−10 *μ*M), LM-031, or licochalcone A (1−100 *μ*M) at 37°C for 24 h, and aggregation was monitored by measuring thioflavin T fluorescence intensity (*n* = 3). *p* values: comparisons between TBP/Q_61_ versus TBP/Q_20_ (^###^*p* < 0.001) or with and without compound addition (^∗^*p* < 0.05, ^∗∗^*p* < 0.01, ^∗∗∗^*p* < 0.001). (c) Filter trap assay of TBP aggregation without or with congo red (10 *μ*M), LM-031, or licochalcone A (100 *μ*M) addition using TBP antibody (*n* = 3). To normalize, the relative trapped TBP/Q_61_ without compound addition is set as 100%. *p* values: comparisons between TBP/Q_61_ versus TBP/Q_20_ (^###^*p* < 0.001) or with and without compound addition (^∗∗∗^*p* < 0.001).

Finally, to examine LM-031 and Licochalcone A in regulating polyQ aggregation, TBP/Q_20-61_ proteins fused to thioredoxin (Trx) and His tags were prepared (Figure 7(a)). Congo red, a potent protein aggregate inhibitor [55], was included for comparison. When aggregate formation was measured with fluorescence generated by thioflavin T binding, significantly increased TBP/Q_61_ aggregation was observed after 1 day's incubation at 37°C as compared to TBP/Q_20_ (501 versus 209 arbitrary unit (AU), *p* < 0.001), which was blocked by congo red (1–10 *μ*M; from 501 to 372–284 AU, *p* = 0.010–<0.001), LM-031 (1–100 *μ*M; from 501 to 286–170 AU, *p* < 0.001), and licochalcone A (10–100 *μ*M; from 501 to 344–291 AU, *p* = 0.002–<0.001) in a concentration-dependent manner (Figure 7(b)). When the protein samples were subjected to filter trap assay and stained with TBP antibody, SDS-insoluble aggregates were evidently reduced in samples treated with congo red (10 *μ*M), LM-031, or licochalcone A (100 *μ*M) (75−60% vs. 100%, *p* < 0.001) (Figure 7(c)), suggesting compounds' direct interference with TBP/Q_61_ aggregate formation.

## 4. Discussion

Up to now, no effective treatments are available for polyQ-induced diseases including SCA17. The revelation of pathogenic mechanisms is the key to the development of therapeutics. In this study, we have shown impaired CREB-dependent transcription and NRF2-ARE pathway and increased AMPK activation in TBP/Q_79_-GFP-expressing SH-SY5Y cells. Our results also demonstrate the neuroprotective effects of licochalcone A and LM-031 on SCA17 SH-SY5Y cell model.

The licochalcone A and five LM compounds displayed a low cytotoxicity in uninduced TBP/Q_79_-GFP 293 and SH-SY5Y cells with IC_50_ ≥ 45 *μ*M as shown in Figure 1. Compound with low cytotoxicity is crucial for its future clinical application to treat diseases. The licochalcone A/LM compounds also demonstrated significant aggregation-inhibitory as well as ROS-reducing effects in TBP/Q_79_-GFP-expressing 293 cells. Among tested LM compounds, LM-031 showed a better aggregation-inhibitory effect than SAHA, indicating its great potential as a therapeutic compound. Such aggregation-inhibitory effect is also found in TBP/Q_79_-GFP SH-SY5Y cells. In addition, LM-031 and licochalcone A displayed neuroprotection via not only rescuing neurite outgrowth deficits but also reducing caspase 3 activity. Further mechanism studies showed that downregulated NRF2, pCREB/CREB, and its downstream genes BCL2 and GADD45B in TBP/Q_79_-GFP SH-SY5Y cells could be upregulated by LM-031/licochalcone A. In addition, increased phosphor/total ratio of AMPK*α* induced by TBP/Q_79_-GFP expression was diminished by LM-031.

In order to know whether LM-031/licochalcone A exerted neuroprotection through acting on CREB, NRF2, and AMPK pathways, knockdown of NRF2 and CREB and AICAR was applied to the SCA17 SH-SY5Y cell model. The effects of reduced aggregation and promoted neurite outgrowth by LM-031/licochalcone A were attenuated by knockdown of NRF2 and CREB. These results indicate that LM-031/licochalcone A protects neurons from neurotoxicity induced by mutant TBP via targeting NRF2 and CREB pathway, both of which are compromised in SCA17.

Several lines of evidence have suggested that targeting NRF2 could be a potential therapeutic strategy for neurodegenerative diseases [56]. Indeed, agents activating NRF2, such as *Gardenia jasminoides*, *Glycyrrhiza inflata*, 3-alkyl luteolin, caffeic acid, and resveratrol are beneficial to different models of polyQ diseases including SCA3, HD, and SBMA [22, 30, 31, 57, 58]. Our study results provide evidence of LM-031 as a new NRF2 activator and its potential for treating SCA17 and other polyQ diseases. However, it should be noted that how LM-031/licochalcone A enhance NRF2 signaling remains to be investigated.

Using the TBP/Q_79_-GFP SH-SY5Y cell model, we showed that pCREB and CREB-mediated gene expression, BCL2 and GADD45B, were downregulated and BAX was upregulated in SCA17. CREB activation is pivotal to neuronal survival and knockdown of CREB leads to progressive neurodegeneration in the hippocampus and striatum, similar to the pathology seen in HD [59]. BCL2 is an antiapoptotic factor and its downregulation leads to decreased cell survival [60]. The knockdown of GADD45B decreases BCL2 and increases proapoptotic factor BAX, suggesting that GADD45B is an intrinsic neuroprotective molecule [61, 62]. Neuronal apoptosis in SCA17 may be attributed to downregulated BCL2 and GADD45B and increased BAX in our study. This result is in consistence with previous reports that impaired CREB-dependent transcription plays an important role in the pathogenesis of polyQ diseases [7]. Several compounds or strategies activating CREB-dependent transcription have been shown to ameliorate pathology and phenotypes of cell and animal models of polyQ diseases including SCA3 and HD [11–16, 63]. Valproic acid can protect against the toxicity of expanded ataxin-3 in an inducible cell model of SCA3 by upregulating CREB-dependent transcription through hyperacetylation [13]. Rolipram provides a neuroprotective effect to R6/2 HD mice via increasing the levels of activated CREB and of BDNF in the striatal spiny neurons [15]. More recently, doxycycline improves neurological deficits and reduces neuropathology in R6/2 mice by enhancing CREB activation in striatum as well as negatively modulating neuroinflammation [16]. However, the effects of CREB-activators have never been shown in SCA17 models. In this study, we demonstrate the neuroprotection effect of a new CREB activator on a SCA17 cell model and its potential for the treatment of polyQ diseases. Nevertheless, it is important to address the limitation of our study that the effects of the compounds tested were only demonstrated in cell models. Future studies using mouse models to show the neuroprotection *in-vivo* is warranted.

AICAR, an AMPK activator, raised pAMPK*α* and diminished the effects of aggregation reducing and neurite outgrowth enhancing by treatment with LM-031, which suggests increased pAMPK*α* contributing to neurotoxicity in SCA17 and LM-031 provides beneficial effect by reducing pAMPK*α*. It is noted that although licochalcone A reduced aggregates and promote neurite outgrowth, it did not attenuate pAMPK*α*, which suggests little effect of licochalcone A on AMPK pathway. Mitochondrial impairment and dysregulation of energy balance are involved in HD pathogenesis. Given that AMPK activation is important in response to energy deprivation, increased mitochondrial biogenesis increased by AMPK activation has been proposed as a potential therapeutic strategy [64–66]. However, studies led by us and other groups suggest that AMPK activation is associated with increased oxidative stress, which may be harmful to neurons and accelerate neurodegeneration [41, 67]. Corrochano and colleagues have shown that endurance exercise is detrimental to HD via AMPK activation in skeletal muscle of HD mice [67]. Studies have shown that AMPK cascades are highly sensitive to oxidative stress [68–70], and a positive feedback interaction between AMPK activation and oxidative stress has been suggested in HD models [41]. In supporting the above implications, Ju et al. showed that an antioxidant, N-acetyl-L-cysteine, reduced the activation and nuclear enrichment of AMPK*α*1 and also increased the levels of BCL2, leading to rescue of neuronal death [41]. In accordance to these results, ours showed that LM-031 decreased aggregation, reduced ROS, and improved neurite outgrowth, which is mediated, at least partially, by reducing AMPK*α* phosphorylation. In contrast, cordycepin reduced aggregates and improved pathological abnormalities by enhancing autophagy, which is mediated through the activation of AMPK in two SCA3 mouse models [71]. Furthermore, activation of AMPK by A769662 and overexpression of an active form of AMPK*α* leads to improved cell viability in HD cell models [72]. Both studies suggest that AMPK activation may contribute to neuronal protection via a mechanism different from the AMPK-ROS pathway. Whether AMPK activation is beneficial or detrimental to polyQ diseases requires future studies to clarify further.

In order to know if the test compounds have chemical chaperone activity, we generated Trx-His-TBP/Q_20−61_ proteins from IPTG-induced bacterial cells and performed thioflavin T binding and filter trap assays and the results showed evidence of the compounds' direct interference with aggregate formation (Figure 7). Whether the upregulated NRF2 and CREB and the downregulated pathway AMPK-ROS are the events subsequent to aggregate inhibition in the SCA17 SH-SY5Y cell model or directly affected by the compounds, or both, warrants future experiments to clarify further.

## 5. Conclusions

**Figure 8:**
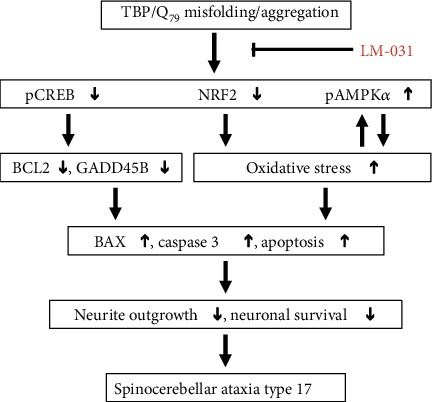
Graphical abstract.

In the present study, we show that NRF2 and CREB pathways are compromised and AMPK is activated, which results in decreased BCL2 and GADD45B and elevated BAX and oxidative stress, and subsequent neurite outgrowth deficits as well as decreased neuronal survival in SCA17 (Figure 8). LM-031 exerts neuroprotective effects in SCA17 cell models by upregulating pCREB and NRF2 and reducing activated AMPK*α* (Figure 8). Given that multiple pathogenic pathways are involved in polyQ including SCA17, LM-031 targeting multiple pathways to provide neuroprotection may have a significant perspective in drug development for SCA17. Given the lack of treatments that prevent disease progression in polyQ-mediated diseases, our study may shed light on the pathogenesis and therapeutic targets in SCA17 and other polyQ diseases. However, future studies in animal models are warranted to confirm our results before applying LM-031 to clinical trials.

## Data Availability

The data used to support the findings of this study are available from the corresponding author upon request.
